# miR-331-3p is involved in glucocorticoid resistance reversion by rapamycin through suppression of the MAPK signaling pathway

**DOI:** 10.1007/s00280-020-04122-z

**Published:** 2020-08-10

**Authors:** Marianna Lucafò, Daria Sicari, Andrea Chicco, Debora Curci, Arianna Bellazzo, Alessia Di Silvestre, Chiara Pegolo, Robert Autry, Erika Cecchin, Sara De Iudicibus, Licio Collavin, William Evans, Giuliana Decorti, Gabriele Stocco

**Affiliations:** 1 Institute for Maternal and Child Health, IRCCS “Burlo Garofolo”, Trieste, Italy; 2 National Laboratory CIB (LNCIB), AREA Science Park, Trieste, Italy; 3 Chemistry, Oncogenesis, Stress, Signaling (COSS), CLCC Eugene Marquis Inserm U1242, University of Rennes-1, Rennes, France; 4 Department of Medicine, Surgery and Health Sciences, University of Trieste, Strada di Fiume 447, 34149 Trieste, Italy; 5 PhD School in Science of Reproduction and Development, University of Trieste, Trieste, Italy; 6 Department of Life Sciences, University of Trieste, Trieste, Italy; 7 Hematological Malignancies Program, St. Jude Children’s Research Hospital, Memphis, TN USA; 8 Experimental and Clinical Pharmacology Unit, Centro di Riferimento Oncologico di Aviano (CRO) IRCCS, Aviano, Italy

**Keywords:** Glucocorticoids, Rapamycin, miRNA, Pharmacoepigenetics

## Abstract

Glucocorticoids (GCs) are commonly used as therapeutic agents for immune-mediated diseases and leukemia. However, considerable inter-individual differences in efficacy have been reported. Several reports indicate that the inhibitor of mTOR rapamycin can reverse GC resistance, but the molecular mechanism involved in this synergistic effect has not been fully defined. In this context, we explored the differential miRNA expression in a GC-resistant CCRF-CEM cell line after treatment with rapamycin alone or in co-treatment with methylprednisolone (MP). The expression analysis identified 70, 99 and 96 miRNAs that were differentially expressed after treatment with MP, rapamycin and their combination compared to non-treated controls, respectively. Two pathways were exclusively altered as a result of the co-treatment: the MAPK and ErbB pathways. We validated the only miRNA upregulated specifically by the co-treatment associated with the MAPK signaling, miR-331-3p. Looking for miR-331-3p targets, MAP2K7, an essential component of the JNK/MAPK pathway, was identified. Interestingly, MAP2K7 expression was downregulated during the co-treatment, causing a decrease in terms of JNK activity. miR-331-3p in mimic-transfected cells led to a significant decrease in MAP2K7 levels and promoted the reversion of GC resistance in vitro. Interestingly, miR-331-3p expression was also associated with GC-resistance in patient leukemia cells taken at diagnosis. The combination of rapamycin with MP restores GC effectiveness through the regulation of different miRNAs, suggesting the important role of these pharmacoepigenetic factors in GC response.

**Electronic supplementary material:**

The online version of this article (10.1007/s00280-020-04122-z) contains supplementary material, which is available to authorized users.

## Introduction

Synthetic glucocorticoids (GCs), such as dexamethasone and methylprednisolone (MP), are commonly used in the treatment of inflammatory and immune diseases, leukemia and in the prevention of rejection in transplant patients [1]. GCs activate glucocorticoid receptor (GR), which, upon nuclear translocation, binds glucocorticoid responsive elements (GREs) resulting in the transcriptional regulation of a large set of target genes [2]. Ligand interaction with GR can also modulate gene expression by the cooperation with other transcription factors, including activator protein-1 (AP-1) and nuclear factor kappa-light-chain-enhancer of activated B cells (NF-kB) [3, 4].

Despite considerable therapeutic utility of GCs, remarkable inter-individual differences in their efficacy have been reported. GC resistance seems to be disease independent: about 10–30% of patients with acute lymphoblastic leukemia [5], inflammatory bowel diseases [6], nephrotic syndrome [7], asthma [8] and rheumatoid arthritis [9] are poor responders to GCs. This variability is due to different mechanisms that include GR mutations, resulting in impaired expression, ligand binding or nuclear translocation, and epigenetic factors [10–12] that however can be in common among the different diseases in which these agents are used. Despite the poor understanding of GC resistance, enhancing GCs efficacy remains important given their widespread clinical use in several diseases.

An increasing number of reports indicate that the immunosuppressive and anti-proliferative agent rapamycin, an inhibitor of mammalian target of rapamycin (mTOR), can reverse GC resistance in different cell types [13–16]. Rapamycin forms a complex with an immunophilin, the 12-kDa FK506-binding protein (FKBP12) that binds and specifically blocks the mTOR complex 1 (mTORC1) activity [17]. Miller and colleagues have demonstrated that, in GC-resistant lymphoid cells, rapamycin reduces the activity of c-Jun N-terminal kinase (JNK), a member of the mitogen-activated protein kinase (MAPK) family, and enhances the apoptosis signaling pathway, revealing the important role of these kinases in GC response [18]. Moreover, the combination of GCs with rapamycin leads to an increase in total GR mRNA and protein, suggesting that the inhibitor of mTOR sensitizes GC resistant cells by modulation of GR expression [13].

Although the main pathways affected in the synergistic effect of rapamycin and GCs have been identified, the molecular mediators involved in the interactions among mTOR, GR and MAPK signaling have not been described yet.

Recent studies have shown that specific non-coding microRNAs (miRNAs) profiles are associated with drug sensitivity, in particular to GCs [19, 20]. miRNAs are small non-coding RNAs able to bind the 3′-UTR of target messenger RNAs (mRNAs), and can regulate expression either post-transcriptionally or post-translationally. In the present study, we investigated the role of miRNAs in the underlying mechanism by which rapamycin sensitizes GC-resistant cells. We also validated the most interesting miRNA, miR-331-3p, in a cohort of pediatric patients with acute lymphoblastic leukemia (ALL), in terms of in vitro resistance to GCs.

## Materials and methods

### Cell cultures

T-lymphoblasts acute lymphocytic leukemia CCRF‐CEM cell line (ATCC, CCL-119) was cultured in RPMI 1640 medium (Sigma-Aldrich, St. Louis, MO, USA, Cat. N. R0883) supplemented with 10% FBS (Euroclone, Pero, MI, Italy, Cat. N. ECS0180L), L-glutamine 2 mM (Euroclone, Pero, MI, Italy, Cat. N. ECB3004D) and antibiotics (Euroclone, Pero, MI, Italy, Cat. N. ECB3001D).

293-T epithelial cells (ATCC, CRL-3216) and U-2 OS epithelial osteosarcoma cells (ATCC, HT9-96) were cultured in DMEM medium (Euroclone, Pero, MI, Italy, Cat. N. ECM0728L) supplemented with 10% FBS (Euroclone, Pero, MI, Italy, Cat. N. ECS0180L) and antibiotics (Euroclone, Pero, MI, Italy, Cat. N. ECB3001D).

H1299 epithelial non-small cell lung cancer cells (ATCC, CRL-5803) were cultured in RPMI medium (Euroclone, Pero, MI, Italy, Cat. N. ECM2001L) supplemented with 10% FBS (Euroclone, Pero, MI, Italy, Cat. N. ECS0180L) and antibiotics (Euroclone, Pero, MI, Italy, Cat. N. ECB3001D).

Cell cultures were maintained according to standard procedures in a humidified incubator at 37 ℃ and with 5% CO_2_, and cell passage was performed once/twice a week. Mycoplasma contamination was evaluated monthly by DNA Hoechst staining and PCR.

### Viability analysis

The effect of methylprednisolone (MP), rapamycin and MP plus rapamycin on cell viability was determined by the colorimetric 3-(4,5-dimethylthiazol-2-yl)-2,5-diphenyl tetrazolium bromide (MTT) (Sigma-Aldrich, St. Louis, MO, USA, Cat. N. M2128) assay. Cells were seeded into 96-well round bottom plates (5 × 10^3^ cells/well) in the presence of MP, rapamycin and with the combination and incubated for 68 h. 20 µl stock MTT (5 mg/mL) were added to each well, and the incubation continued for additional 4 h. Cells were lysed with DMSO. Absorbance was measured at 540 nm using a microplate reader (Automated Microplate Reader EL311, BIOTEK^®^ Instruments, Vermont, USA). All measurements were done in six replicates, and at least three independent experiments were carried out.

### Total RNA isolation

Total RNA was extracted using TRIzol reagent (Thermo Scientific, Waltham, MA USA, Cat. N. 15596026) according to manufacturer’s instructions. The RNA concentration and purity were calculated by Nano Drop instrument (NanoDrop 2000, EuroClone, Milan, Italy).

### miRNA expression profile analysis

To evaluate miRNA expression profile, TaqMan^®^ Low Density Array Human MicroRNA A Card v2.0 (Applied Biosystems™, Carlsbad, California, USA, Cat. N. 4398965) containing 377 different probes (https://www.thermofisher.com/order/catalog/product/4398965#/4398965) was used. The arrays were processed and analyzed in accordance to manufacturer's protocols. PCR amplification was performed using TaqMan miRNA reverse transcription kit and Megaplex RT Primers and the resulting cDNA combined with TaqMan Universal PCR Master Mix (Applied Biosystem, Foster City, CA, USA). The experiment was repeated three times. Data were normalized using the Small Nucleolar RNA C/D Box 44 (RNU44) as endogenous control. The comparative Ct method (2^− ΔΔCT^) was used to evaluate the relative changes in miRNA expression. A cut off of relative expression (RE) > 1.5 for up-regulated miRNAs and < 0.5 for down-regulated miRNAs was established.

### MiRNA pathway analysis and identification of putative miRNA targets

For the identification of the networks and pathways of the differentially expressed miRNAs, we used DIANA miRPath (v3.0) [21]. TargetScan database [22] and miRDIP software (https://ophid.utoronto.ca/mirDIP/) were then used to predict miRNA targets (in 3′-UTR regions).

### Validation of miR-331-3p expression by qRT-PCR

Expression levels of the candidate miR-331-3p were evaluated by real-time RT-PCR TaqMan^®^ analysis (Applied Biosystems™, Carlsbad, California, USA, Cat. N. 4427975 ID: 000545) using the CFX96 real-time system-C1000 Thermal Cycler (Bio-Rad Laboratories, Hercules, CA, USA). The reverse transcription reaction was carried out with the Taqman MicroRNA Reverse Transcription Kit (Life Technologies, Carlsbad, California, USA, Cat. N. 4366596) according to manufacturer's instructions and the real-time PCR was performed in triplicate using the Taqman MicroRNA assay. The expression levels of the selected miRNA were determined using the comparative Ct method (2^−ΔΔCT^ method). miR-331-3p expression values were normalized using RNA U6 Small Nuclear 1 (U6) (Applied Biosystems™, Carlsbad, California, USA, Cat. N. 4427975 ID: 001973) as reference gene.

### TaqMan gene expression analysis

Expression levels of NR3C1 and GILZ were evaluated by TaqMan^®^ Gene Expression Assay (Applied Biosystems™, Carlsbad, California, USA, Cat. N. 4331182 ID: Hs00353740_m1, Hs00608272_m1,) and the reverse transcription reaction was carried out with the High Capacity RNA-to-cDNA Kit (Applied Biosystem, Foster City, CA, USA). The expression levels were determined using the comparative Ct method (2^− ΔΔCT^ method). Gene expression values were normalized using 18S ribosomal RNA (18S) (Applied Biosystems™, Carlsbad, California, USA, Cat. N. 4331182 ID: Hs99999901_s1) as reference gene.

### Western blot

Cells (2 × 10^6^) were treated with MP, rapamycin and in combination for 72 h; total cell extracts were prepared in RIPA buffer without SDS (150 mM NaCl, 50 mM Tris–HCl pH8, 1 mM EDTA, 1% NP-40, 0.5% Na-deoxycholate) supplemented with 1 mM PMSF, 5 mM NaF, 1 mM Na_3_VO_4_, 10 µg/ml CLAP. Protein concentration was determined with Bio-Rad Protein Assay Reagent (Bio-Rad Laboratories, Hercules, CA, USA, Cat. N. 500-006). Western blot experiments were performed according to standard procedures. Lysates were resolved by SDS/PAGE and transferred to nitrocellulose membranes (Sigma-Aldrich, St. Louis, MO, USA, Cat. N. GE 10600002). Western blot analysis was performed according to standard procedures using the following primary antibodies: anti-p-JNK Thr183/Tyr185 (Cell Signaling, Beverly, MA, USA, Cat. N. s9251), anti-JNK (Santa Cruz, Dallas, Texas, USA, Cat. N. sc-6254), anti-HSP90 (Santa Cruz, Dallas, Texas, USA, Cat. N. sc-13119), anti p-GR Ser 211 (Cell Signaling, Beverly, MA, USA, Cat. N. s4161), anti-GR E-20 (Santa Cruz, Dallas, Texas, USA, Cat.N. sc-1003), anti-actin (Sigma-Aldrich, St. Louis, MO, USA, Cat. N. A2066), anti-MAP2K7 (Abcam, Cambridge, UK, Cat. N. ab52618). Anti-mouse and anti-rabbit HRPO-conjugated (Abcam, Cambridge,UK, Cat. N. ab214879 and ab214880) were used as secondary antibodies.

### Transfections and mimic assay

293-T (4 × 10^5^), U-2 0S (2 × 10^5^) and H1299 (1 × 10^5^) cells were seeded 24 h prior to transfection. Cells were transfected with Lipofectamine RNAiMax (Invitrogen, Carlsbad, California, USA, Cat. N. 13778-150), following manufacturer’s instructions. Briefly, 3 µl Lipofectamine RNAiMax for each experimental point, miR-331-3p mimic (Thermo Scientific, Waltham, MA, USA, Cat. N. 4464066 Assay ID: MC10881), at a final concentration of 3 nM, or All Star Negative control (Qiagen, Gaithersburg, MD, USA, Cat. N. 1027281), used as control, at the final concentration of 3 nM, were added in 300 µl Opti-MEM Medium without serum (Thermo Scientific, Waltham, MA, USA, Cat. N. 11058021). After 20 min incubation at room temperature, RNAiMAX-RNA molecules mix was added to cells, plated in medium without antibiotics. The day after, culture medium was replaced with culture medium supplemented with antibiotics. Cells were harvested 48 h after transfection, according to the manufacturer’s guidelines.

### Luciferase assay

293-T cells (5 × 10^4^), plated in 24-well plates, were firstly transfected with Lipofectamine RNAiMax (Invitrogen, Carlsbad, California, USA, Cat. N. 13778-150), following manufacturer’s instructions. Briefly, 1 µl Lipofectamine RNAiMax for each experimental point, miR-331-3p mimic (Thermo Scientific, Waltham, MA, USA, Cat. N. 4464066 Assay ID: MC10881), at a final concentration of 3 nM, siRNA targeting MAP2K7 (5′-AGUCACGAUUAGCUGCUUG-3′, Eurofins Genomics, Ebersberg, Germany) at a final concentration of 3 nM, or All Star Negative control (Qiagen, Gaithersburg, MD, USA, Cat. N. 1027281), used as control, at the final concentration of 3 nM, were added in 100 µl Opti-MEM Medium without serum (Thermo Scientific, Waltham, MA, USA, Cat. N. 11058021). After 20 min incubation at room temperature, RNAiMAX-RNA molecules mix was added to cells, plated in medium without antibiotics. The day after, the pEZX-MT06 dual-luciferase vector containing the 3′-Untraslated Region (3′-UTR) of MAP2K7 (Genecopoeia, Rockville, MD, 20850, USA, Cat. N. HmiT117977-MT06) or the control empty vector (Genecopoeia, Rockville, MD, USA, Cat.N. CmiT000001-MT06) were transiently transfected into 293-T cell using the Lipofectamine 3000 reagent (Invitrogen, Carlsbad, California, USA, Cat. N. L3000015) following manufacturer’s instructions. Briefly, 0.5 µl Lipofectamine 3000 and 0,4 µl of P3000, for each experimental point, and the plasmid vectors at a final concentration of 200 ng were added in 50 µl Opti-MEM Medium without serum (Thermo Scientific, Waltham, MA, USA, Cat. N. 11058021). After 20 min incubation at room temperature, Lipo 3000-DNA molecules mix was added to cells. Twenty-four hours later, Luciferase assay was performed using Dual Luciferase Reporter Assay System (Promega, Wisconsin, USA, Cat. N. E1910): luciferases activity was measured on a Promega luminometer (Promega), following manufacturer’s instructions.

### miR-331-3p levels and GC sensitivity in ALL patient samples

MiR-331-3p expression data and LC50 values were downloaded from the Gene Expression Omnibus (GEO) (https://www.ncbi.nlm.nih.gov/geo/), in particular from the project GSE76849 and GSE66705 [23, 24]. Written, informed consent and assent following Institutional Review Board, NCI, FDA, and Office for Human Research Protections Guidelines was obtained and described in Paugh et al. [24]. The probe “42887” was used for evaluating miR-331-3p expression in the miRCURY LNA 10.0 microarray. Background subtracted minimum translated data were log2 transformed and then quantile normalized prior to statistical analysis. The microRNA expression data are available for download via https://trident.stjude.org, and https://www.stjuderesearch.org/evans/. The GC LC50 of patient-derived leukemia cells taken at initial diagnosis from bone marrow aspirate or peripheral blood was determined with the use of the 4-day in vitro MTT drug-resistance assay [24]. Glucocorticoid resistant ALL was defined as having an LC50 of 64 µM or greater, whereas glucocorticoid sensitive cases were defined as having an LC50 less than 0.1 µM.

### Statistical analysis

Data are represented as mean ± standard error (SE). Statistical analyses were performed using Graph-Pad Prism version 4.00 (GraphPad, La Jolla, CA, USA). Two-way and one-way ANOVA with Bonferroni post-test and t test were used for the analysis of inhibition of proliferation and expression. *p* values < 0.05 were considered statistically significant. For the analysis evaluating an association between miR expression and MP sensitivity in patient samples, a linear model with formula log10 (MP LC50) ~ log2 (miRNA expression) was used.

## Results

### Sensitivity to MP, rapamycin and to co-treatment

**Fig. 1:**
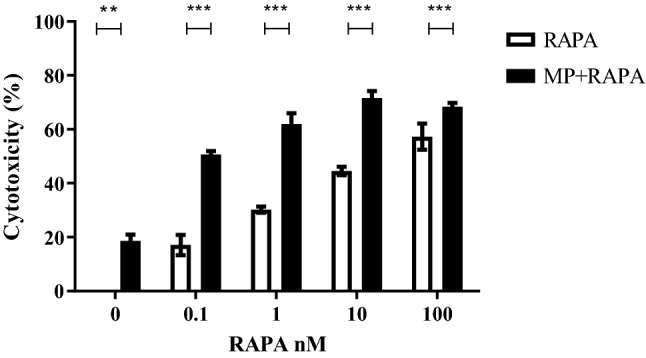
Cytotoxic effect of rapamycin alone (RAPA; range from 0.1 to 100 nM) and in combination with methylprednisolone (MP 50 µM) on CCRF-CEM cells. Cells were exposed for 72 h to drugs and cytotoxicity was evaluated by the MTT assay. The value is the percentage of treated cells vs untreated controls. Results are mean values ± SE from three independent experiments; two-way ANOVA (*p* < 0.0001) and Bonferroni post-test ***p value < 0.001

To evaluate the role of rapamycin in sensitizing cells to MP, we firstly measured cell viability by MTT cytotoxicity assay. CCRF-CEM cells were treated for 72 h with MP alone (concentration 50 µM), with rapamycin (concentrations 0.1–100 nM) alone or in combination with MP. As shown in Fig. 1, even if MP alone seems to have a significant effect on the cell viability, a more significant increase in cytotoxicity was observed in cells treated with rapamycin in combination with MP in comparison with rapamycin alone, supporting the hypothesis that rapamycin may improve the efficacy of GCs.

Based on these results, the concentration (1 nM) of rapamycin with an intermediate effect in combination with MP was chosen for further experiments.

### Evaluation of GR expression and transactivation

To verify whether the ability of rapamycin to restore the sensitivity to GCs was dependent on the expression of the GR and its ability to promote GC-induced leucine zipper (GILZ) transcription, we performed western blot and qRT-PCR experiments on CCRF-CEM cells treated with MP, rapamycin and in co-treatment.

**Fig. 2:**
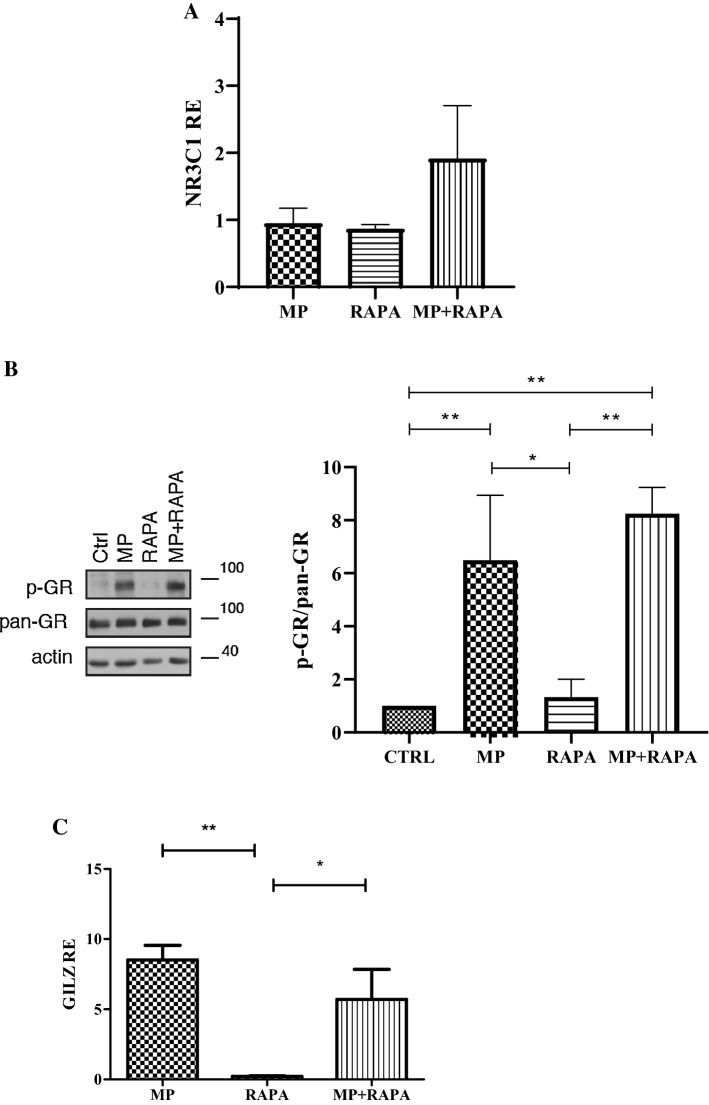
Effect of MP and rapamycin on GR protein levels and its transcriptional activity. **a** Relative expression (RE) of NR3C1 after incubation for 72 h with MP, RAPA and MP + RAPA. One-way ANOVA (*p* = ns). **b** Protein levels of GR in CCRF-CEM in untreated cells (CTRL) and after treatment with MP (50 µM), rapamycin (RAPA 1 nM) and in combination (MP + RAPA). **c** Relative expression (RE) of GILZ after incubation for 72 h with MP, RAPA and MP + RAPA. One-way ANOVA (*p* = 0.004) and Bonferroni post-test **p value < 0.001; *p value < 0.05. The data are reported as means ± SE of three independent experiments performed in triplicate

Interestingly, we did not observe any significant change in GR mRNA (NR3C1 gene; Fig. 2a) and protein levels (total GR (pan-GR)) (Fig. 2b) after exposure to the drugs. GR phosphorylation status changed only in the presence of its ligand but no significant difference was detected between the treatment with MP alone and the co-treatment (Fig. 2b).

As expected, a strong upregulation of GILZ was observed after treatment with MP alone (Fig. 2c). Co-treatment with rapamycin did not further increase the transactivation of the GC target gene (Fig. 2c) suggesting that the increased cytotoxicity observed during the co-treatment is not related to a higher transcriptional activity of the GR.

### Differentially expressed miRNAs

To understand the effects of MP, rapamycin and their combination on miRNAs expression profile, we performed a large-scale analysis of 377 miRNAs on CCRF-CEM cells treated for 72 h compared to untreated cells.

The expression analysis identified 70, 99 and 96 miRNAs that were differentially expressed after treatment with MP (50 µM), rapamycin (1 nM), and their combination, respectively (Online Resource Fig. 1 and Online Resource Table1).

### Pathways affected by differentially expressed miRNAs

Next, we performed a pathway enrichment analysis of protein-coding genes potentially targeted by the identified miRNAs using DIANA algorithm and TargetScan databases containing predicted miRNA-target interactions.

Many cancer and metabolism related networks were found to be involved after treatment with the drugs but only two pathways were altered by the combination treatment and not by MP or rapamycin alone: the MAPK and the epidermal growth factor receptor (ErbB) signaling pathways (Table 1). Ninety-eight genes related to the MAPK signaling pathway were predicted targets of 41 upregulated miRNAs, while 22 genes related to the ErbB signaling pathway turned out to be potentially targeted by the 14 miRNAs downregulated by MP and RAPA co-treatment (Online Resource Table 2).

**Table 1:** Pathways enriched in genes targeted by the differentially regulated miRNAs; methylprednisolone (MP), rapamycin (RAPA)

Upregulated miRNAs									
Pathways	MP			RAPA			MP + RAPA		
	*p* value	Gene	miRNA	*p* value	Gene	miRNA	*p* value	Gene	miRNA
ECM-receptor interaction	5.067 e-27	26	14	1.611 e-27	30	20	1.674 e-21	36	21
Biotin metabolism	7.131 e-5	2	2	0.0009	2	3	0.002	2	3
Vitamin B6 metabolism	0.00057	4	4	0.0294	3	3	0.0416	3	4
PI3K-Akt signaling pathway	0.00687	96	29	0.0009	124	44	0.0025	130	48
p53 signaling pathway	0.0086	24	18	0.0071	30	27	0.00271	33	31
Ras signaling pathway	0.0201	64	26	0.003	85	39	0.027	83	42
Neurotrophin signaling pathway	0.042	36	22	0.0294	47	31	0.0053	53	34
Glycosaminoglycan biosynthesis	0.025	6	5	–	–	–	0.0065	11	11
Fatty acid biosynthesis	–	–	–	1.898 e-13	4	4	3.734 e-12	4	5
Ubiquitin mediated proteolysis	–	–	–	0.0071	55	33	0.0146	57	37
Prion diseases	–	–	–	5.169	9	12	–	–	–
Bacterial invasion of epithelial cells	–	–	–	0.0052	34	28	–	–	–
Estrogen signaling pathway	0.0291	29	16	–	–	–	–	–	–
Protein digestion and absorption	0.02	31	15	–	–	–	–	–	–
Tight junction	0.028	41	21	–	–	–	–	–	–
Proteoglycans in cancer	0.019	74	39	–	–	–	–	–	–
MAPK signaling pathway	–	–	–	–	–	–	0.00576	98	41

Downregulated miRNAs									
Pathways	MP			RAPA			MP + RAPA		
	*p* value	Gene	miRNA	*p* value	Gene	miRNA	*p* value	Gene	miRNA
Fatty acid biosynthesis	3.31 e-26	4	3	1.123 e-23	3	2	7.532 e-21	3	1
Fatty acid metabolism	2.069 e-8	11	9	0.00064	9	8	0.007	9	6
Proteoglycans in cancer	3.569 e-5	40	16	0.00074	37	20	0.0019	35	15
N-Glycan biosynthesis	0.0314	10	9	0.0248	12	9	0.03	10	8
Pathway regulating pluripotency of stem cells	0.005	30	15	0.03099	30	20	–	–	–
Glycosphingolipid biosynthesis	0.0106	5	3	0.0248	6	7	–	–	–
PI3K-Akt signaling pathway	–	–	–	0.0248	67	22	–	–	–
Acute myeloid leukemia	–	–	–	0.0309	15	14	–	–	–
Thyroid hormone signaling pathway	0.0015	20	15	–	–	–	–	–	–
Thyroid hormone synthesis	0.0075	12	8	–	–	–	–	–	–
ErbB signaling pathway	–	–	–	–	–	–	0.014	22	14

Among the 41 upregulated miRNAs linked to the MAPK signaling pathway, only 1 was exclusively deregulated by the co-treatment but not by the treatments with RAPA or MP alone, the miR-331-3p (Online Resource Table 1). Therefore we chose this miRNA as involved in the potential molecular mechanism of the synergic cytotoxicity observed during the co-treatment.

**Fig. 3:**
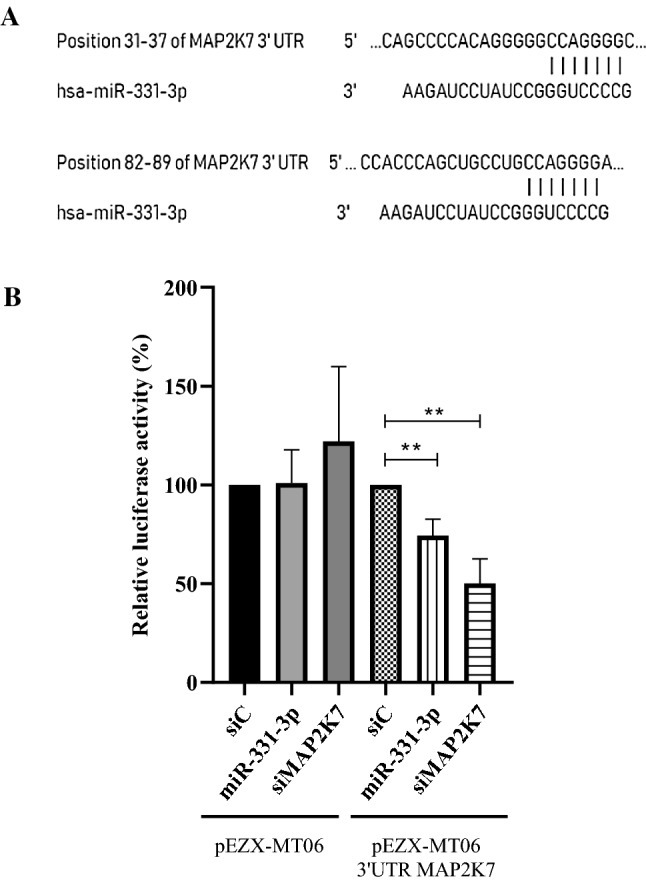
**a** Predicted consequential pairing of MAP2K7 target region (top) and miR331-3p (bottom). **b** Luciferase reporter assays was performed after 293 T cells were transfected with MAP2K7 3′-UTR-reporter constructed together with miR-331-3p mimics or negative controls (siC). In parallel, cells were transfected with the empty vector together with miR-331-3p mimics, siRNA targeting MAP2K7 3′-UTR or siC. *T*-test analysis **p value < 0.01. The data are reported as means ± SE of three independent experiments performed in triplicate

A list of putative target genes of miR-331-3p, predicted by Diana miRPath software, belonging to the MAPK signaling pathway was obtained (Online Resource Table 3). TargetScan Human analysis (release 7.2) was performed on all these identified putative target genes and only for MAP2K7 it was predicted that the 3′-UTR contains more than one putative miR-331-3p binding sites; in particular two sites were predicted, in position 31–37 and 82–89 (Fig. 3a).

The first target site had a context score of 40% on TargetScan, the second site had a higher score of 87%, and is more conserved within the seed region. For this reason, miR-331-3p and its putative target MAP2K7, an upstream regulator of JNK proteins, were selected for further investigations.

A list of miR-331-3p putative target genes were also obtained using another web tool called microRNA Data Integration Portal (miRDIP; Online Resource Table 4).

### MAP2K7 is a direct target of miR-331-3p

To verify the hypothesis, luciferase reporter vector containing 3′UTR of MAP2K7 in 293-T cells co-transfected with miR-331-3p mimic or siRNA targeting MAP2K7 3′UTR (positive control) were used. As shown in Fig. 3b, the relative luciferase activity was significantly downregulated by miR-331-3p in comparison with the negative control (siC). As expected, no differences were observed using the empty vector in 293-T cells co-transfected as described above.

### qRT-PCR validation data

**Fig. 4:**
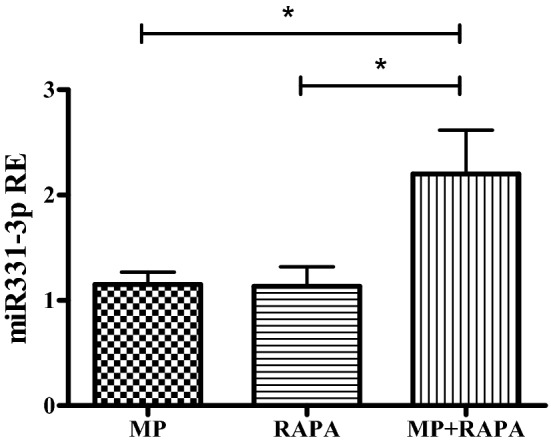
Relative expression (RE) of miR-331-3p after treatment with MP (50 µM), RAPA (1 nM) and MP + RAPA for 72 h. One-way ANOVA (*p* = 0.023) and Bonferroni post-test *p value < 0.05. The data are reported as means ± SE of three independent experiments performed in triplicate

We first validated the selective up-regulation of miR-331-3p after the co-treatment by qRT-PCR, in CCRF-CEM cells treated with MP, rapamycin and their combination for 72 h. As reported in Fig. 4, results confirm its upregulation after the co-treatment.

### Quantification of MAP2K7 and JNK activation

**Fig. 5:**
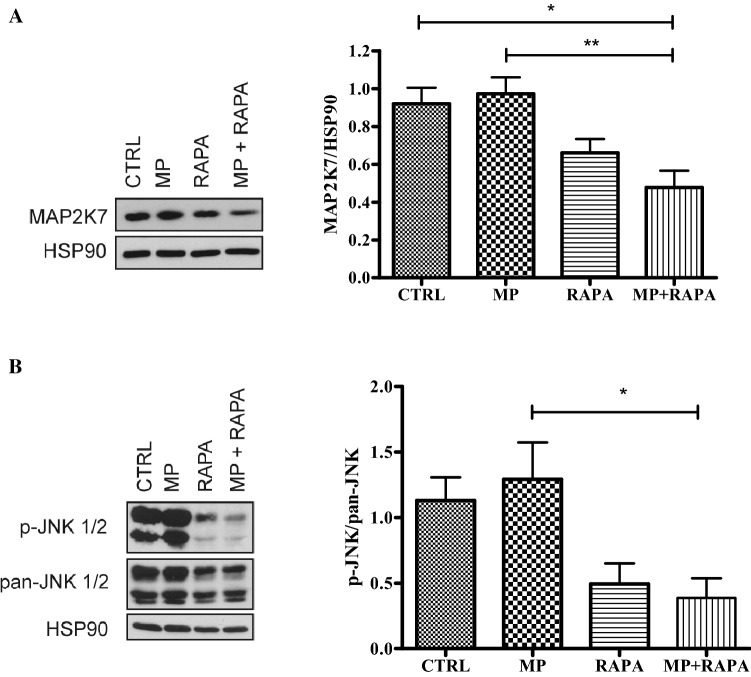
**a** Protein expression of MAP2K7 by western blot analysis on CCRF-CEM cells untreated (CTRL) treated with MP (50 µM), RAPA (1 nM) and MP + RAPA for 72 h. MAP2K7 expression evaluated in cells treated and untreated for 72 h normalized by HSP90 protein; one-way ANOVA analyses: *p* = 0.0022, Bonferroni’s post-test *p value < 0.05; **p value < 0.01. The data are reported as means ± SE of three independent experiments performed in triplicate. **b** Protein expression of p-JNK and pan-JNK by western blot analysis on CCRF-CEM cells untreated (CTRL) treated with MP (50 µM), RAPA (1 nM) and MP + RAPA for 72 h. P-JNK expression evaluated in cells treated and untreated for 72 h in respect to pan-JNK protein. One-way ANOVA analyses: *p* = 0.0022, Bonferroni’s post-test *p value < 0.05; **p value < 0.01. The data are reported as means ± SE of three independent experiments performed in triplicate.

The protein expression of MAP2K7 in CCRF-CEM cells after treatment with MP, rapamycin alone or in co-treatment for 72 h was measured by western blot. The results showed a decrease in the protein level during the co-treatment (Fig. 5a).

To evaluate whether the combination of rapamycin and MP could inhibit JNK activation, through reduced MAP2K7 expression, we measured phospho-JNK (p-JNK) and total JNK (pan-JNK) levels in CCRF-CEM human leukemia cell line post-treatment by western blot. As shown in Fig. 5b, a significant difference among all the groups analyzed was observed; in particular, the results demonstrated a significant decrease in the phosphorylated active form p-JNK after treatment with rapamycin (p-JNK/pan-JNK = 0.49) and in co-treatment with MP (p-JNK/pan-JNK = 0.38). These data confirm the hypothesis that the effects observed during co-treatment could be caused by the inhibition of JNK activation following a reduction of MAP2K7 protein levels.

### miR-331-3p reduces both MAP2K7 and p-JNK and sensitizes GC resistant cells to MP

**Fig. 6:**
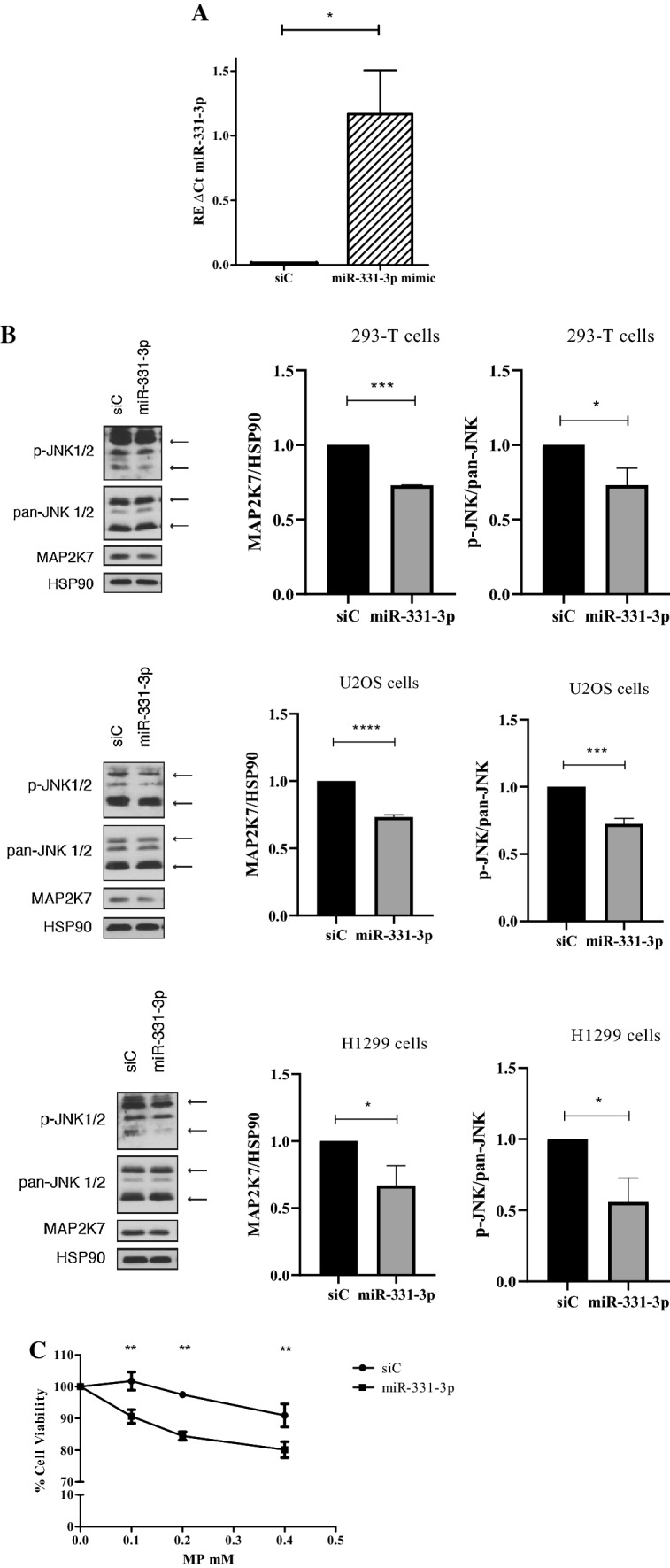
**a** Relative expression (RE) of miR-331-3p in 293-T cells transfected with the miR-331-3p mimic (miR-331) or the scramble control (siC). T-test analyses: *p value < 0.05. The data are reported as means ± SE of three independent experiments performed in triplicate. **b** Protein expression of MAP2K7, p-JNK and pan-JNK by western blot analysis on 293-T, U-2 OS and H1299 cells transfected with the miR-331-3p mimic (miR-331) or the scramble control (siC). *T*-test analyses: ***p < 0.001, ****p < 0.0001. The data are reported as means ± SE of three independent experiments performed in triplicate. C) Effect of MP on 293-T cell viability in cells transfected with miR-331-3p mimic (miR-331-3p) and control (siC). Two-way ANOVA p < 0.0001 and Bonferroni post-test **p < 0.01. The data are reported as means ± SE of three independent experiments

To verify that miR-331-3p targets MAP2K7 directly, we designed gain-of-function experiments of miR-331-3p in the 293-T, U-2 OS and H1299 cell lines. We forced the expression of miR-331-3p in cells using miR-331-3p mimic, and miRNA levels were increased significantly with respect to the scramble control (Fig. 6a).

As shown in Fig. 6b, the miR-331-3p in mimic-transfected cells led to a significant decrease in MAP2K7 protein levels. The effect of miR-331-3p mimic was tested also on p-JNK protein expression. As shown, the phosphorylation of JNK decreased after transfection with the miR-331-3p mimic, confirming its role in the regulation of MAP2K7 (Fig. 6b).

To investigate on the role of miR-331-3p in the reversion of GC resistance, mimic-transfected cells were treated with different concentrations of MP (concentrations 0.1–0.4 mM) for 72 h and the cytotoxicity was evaluated by MTT assay. The results clearly showed that cell viability was lower in miR-331-3p transfected cells treated with MP (Fig. 6c). No difference was observed after transfection of mimic miR-331-3p alone or siRNA control alone on cell growth.

### miR-331-3p as a marker of GC resistance in pediatric ALL patients

To investigate the association between the miR-331-3p levels and the GC sensitivity in ALL patients, the miRNA expression values (available in Gene Expression Omnibus (GEO; https://www.ncbi.nlm.nih.gov/geo/) from the project GSE76849) were compared to sensitivity/resistance data (available in GEO from the project GSE66705). The GC LC50 of patient-derived leukemia cells taken at initial diagnosis from bone marrow aspirate or peripheral blood was determined with the use of the 4-day in vitro MTT drug-resistance assay [24]. GC resistant ALL was defined as having an LC50 of 64 μM or greater, whereas glucocorticoid sensitive cases were defined as having an LC50 less than 0.1 μM.

**Fig. 7:**
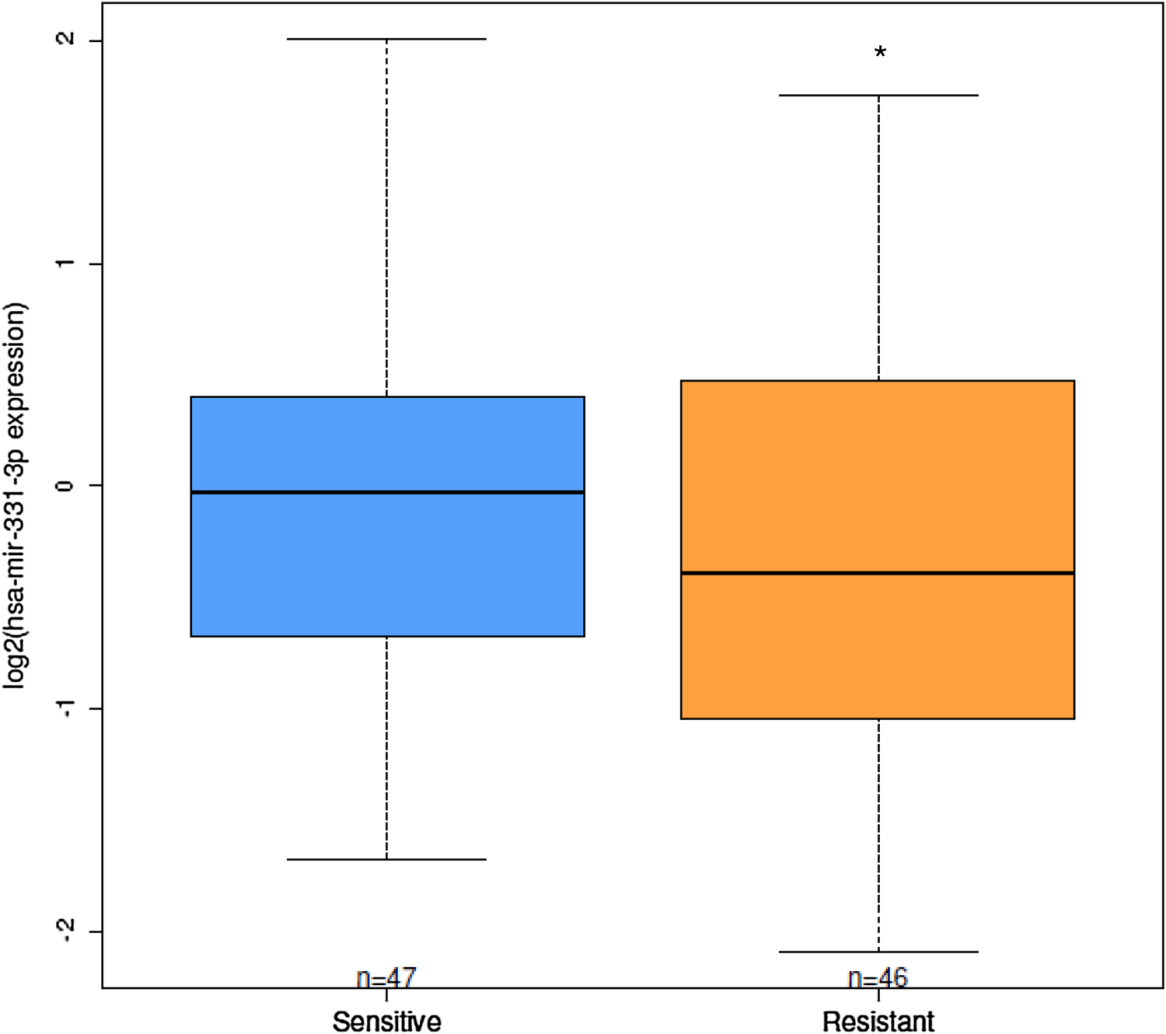
Expression level of miR-331-3p in leukemia cells derived from patients sensitive or resistant to GCs. Linear model *p < 0.05

Among the 93 enrolled patients, 46 were steroid resistant and 47 steroid sensitive. Steroid sensitive patients presented significantly higher miR-331-3p in comparison with steroid resistant group (*p* = 0.044; Fig. 7). Moreover, among the 41 miRNAs upregulated by the cotreatment in vitro*,* linked to the MAPK signaling pathway, in addition to miR-331-3p, other 5 miRNAs (miR-222-3p, miR-221-3p, miR-339-5p, miR-149-5p and miR-15a-5p) showed differential expression in steroid sensitive patients respect to the steroid resistant group (Online Resource Table 5).

## Discussion

GC resistance is a major driver of therapeutic failure in immune-mediated diseases. Considering the large number of reports that describe the effect of the mTOR inhibitor rapamycin in reverting GC resistance in vitro [13, 15, 18], in this study we investigated the involvement of miRNAs in the underlying molecular mechanism of GC sensitivity and how it is restored by rapamycin co-treatment. miRNAs involvement in the modulation of drug response is of great interest to the scientific community but studies about this topic are still very limited, and the possible correlation between miRNAs expression and variability of GC response has been poorly examined [19].

We show that rapamycin can reverse resistance to MP, one of the most commonly used steroids for the management of several diseases, in our GC-resistant cell model, the CCRF-CEM human leukemia cell line. In our model system, results demonstrate that GR levels, in terms of mRNA and protein, were unaffected by the single treatments or by the co-treatment. These data seem to be in contrast with the previously published paper in which authors suggested that rapamycin increased GC-induced apoptosis mainly through a significant upregulation of the GR protein [13]. The differences observed could be due to the experimental conditions: in particular, Guo and collaborators used a different cell line, selected for GC resistance, and dexamethasone instead of MP.

Moreover, the upregulation of the GR target gene GILZ, induced upon GC stimulation, resulted to be similar after treatment with MP used alone or in combination with rapamycin, again suggesting that rapamycin could enhance GC efficacy independently of GR activity. This hypothesis was confirmed evaluating the GR phosphorylation status, given that transactivation function strongly depends on this post-translational modification [25].

Emerging evidence indicates that miRNAs could be involved in GCs resistance [26, 27], therefore in the present study we implemented a miRNA array analysis to detect the differential expression profile of miRNAs in CCRF-CEM cells after treatment with MP, rapamycin and in co-treatment. Among all human miRNAs analyzed, 70, 99 and 96 were differentially expressed after treatment with MP, rapamycin and the combination, respectively.

Some of the miRNAs identified as deregulated after treatments with the GC and rapamycin have previously shown a similar trend in leukemic cells, such as for miR185-5p and miR-221 [28, 29]. In particular, in our experiments, the fold change of miR-185-5p increased from 7.86 during MP treatment up to 12.45 during the co-treatment with rapamycin, confirming the importance of this miRNA in GC response. Indeed, Chen and collaborators have reported that the overexpression of miR-185-5p significantly enhanced GC sensitivity in CEM-C1 cells (GC resistant) by increasing the rate of cell cycle arrest and apoptosis, and decreasing survival, accompanied by a decrease in mTORC activity [28]. Concerning the role of miR-221, Kotani and colleagues have shown that miR-128b acts in a cooperative manner with miR-221, increasing GC effect in a leukemic cell line [29]. In our experiments, the expression levels of miR-221 was upregulated by the three pharmacological conditions, and in particular after the co-treatment, however no difference were observed in terms of expression of miR-128b. Moreover, miR-181a-5p, miR-20a-5p and miR-139-5p target MCL1 [30–32], a protein involved in the mechanism of GC-resistance reversion induced by rapamycin [16].

Pathway enrichment analysis of differentially expressed miRNAs revealed that two biological processes were altered specifically by the co-treatment: the MAPK and the ErbB signaling pathways.

Since several reports support the hypothesis that MAPKs influence GC sensitivity [18, 33, 34], we further analyzed 41 miRNAs associated to the MAPK signaling pathway, and found that only one was deregulated by the co-treatment but not by the single treatments (both rapamycin and MP), miR-331-3p. Currently, the molecular mechanism by which miR-331-3p could be regulated by rapamycin or the GC has never been investigated. Preliminary evidence has documented the biological function of the circular RNA circ_0001649 as sponge for the regulation of miR-331-3p expression [35] but no data have been reported about the ability of GCs or rapamycin to modulate the levels of this circular RNA.

To explore the mechanism by which miR-331-3p regulates the GC response, we evaluated potential targets of miR-331-3p, identifying MAP2K7 as the main putative target. The hypothesis was confirmed by the luciferase reporter assay which showed that miR-331-3p directly targeted the MAP2K7 3′-UTR.

MAP2K7, also known as MKK7, specifically activates JNK which, in turn, modifies the activity of numerous proteins by phosphorylation [33, 36]. Moreover, it is known that activated MAP2K7 enhances the proliferation, metastasis and progression of cancer [37].

In our experiments, rapamycin in combination with MP reduces the expression of MAP2K7, possibly through the upregulation of miR-331-3p. We analyzed also the expression levels of its downstream regulator, the JNK protein. The results demonstrated a significant decrease in the phosphorylated active form p-JNK after co-treatment in comparison to the MP treatment alone, confirming again the key role of MAP2K7 in the sensitization to MP caused by rapamycin. However, rapamycin also reduced p-JNK even if the difference with MP is not significant. It is known that p-JNK can be regulated also by MAP2K4 therefore the reduction observed after rapamycin treatment could be related to the modulation of this protein. JNKs, also called stress activated protein kinases, are members of the MAPK family implicated in the regulation of a wide range of cellular functions [38]. The JNKs phosphorylate and activate members of AP-1 transcription family as well as other substrates involved in numerous biological functions, such as apoptosis, cytoskeletal rearrangement, transcriptional activation and protein ubiquitination [39]. Miller and colleagues have already demonstrated that, in GC-resistant lymphoid cells, rapamycin diminishes JNK activity and enhances apoptosis signaling pathway, revealing the important role of these proteins in GC response [18]. Moreover, sensitivity to GCs could be restored by co-treatment with JNK inhibitors in HeLa cells [40].

On the contrary, few papers have described a direct crosstalk between GCs and MAP2K7. For instance, Szatmáry and collaborators observed that the activation of endogenous JNK by its specific activator MAP2K7 inhibited GR-mediated transcriptional activation [41]. In addition, Bruna and collaborators analyzed the negative regulation of MAPK pathways by GCs and found that treatment with dexamethasone inhibited MAP2K7-induced activation of JNK by the GR-mediated sequestration of the protein [42]. In both cases, the cellular models used were HeLa cells, a non-lymphoid GC sensitive cell line; this could, at least in part, explain the different behavior observed for the GR in our experiments, in which its expression and activation were not altered.

As hypothesized, a forced expression of miR-331-3p decreased MAP2K7 expression levels, confirming that this protein is a target of miR-331-3p. Importantly, miR-331-3p could revert GC resistance, as indicated by the decreased rate of cellular viability; however, further analysis on lymphoid cells transfected with the mimic should be performed to better appreciate an increased effect on MP cytotoxicity.

The miR-331-3p gene is located at 12q22 and has been found to regulate the development and progression of various types of cancer cells [43–45]; in particular, miR‑331-3p contributes to cell growth regulation by targeting ERBB expression and blocks phosphatidylinositol 3-kinase (PI3K)/AKT signaling, one of the upstream pathways of JNK signaling. Since miR-331-3p is involved in regulating PI3K/Akt and JNK signaling networks, the identification of small molecules that could increase expression of miR-331-3p or the use of its miRNA mimics could represent new strategies to overcome the resistance due to PI3K-Akt-JNK activation.

Interestingly miR-331-3p expression was associated with GC resistance evaluated ex vivo in a cohort of patients with pediatric ALL: patients with higher miRNA levels presented increased sensitivity to MP, an observation consistent with the contribution of this miRNA in modulating GC response.

## Conclusions

Our study is the first that highlights a role of miR-331-3p in the mechanism of GC resistance reversion by rapamycin and demonstrates that miR-331-3p directly targets MAP2K7.

Although the role of all the other miRNAs differentially expressed during the co-treatment should be further evaluated, our data support the hypothesis that treatment with MP plus rapamycin modulates the MAPK pathway through upregulation of miR-331-3p.

Considering data obtained on ALL patients, in the future it could be interesting to measure miR-331-3p levels in other clinical cancer samples, to evaluate if this miRNA could be a potential marker of patient response to GC treatment and/or a therapeutic target useful for the development of new pharmacological approaches to restore GC sensitivity.

## Electronic supplementary material

Below is the link to the electronic supplementary material.

Supplementary Online Resource Fig.1 (DOCX 105 kb)

Supplementary Online Resource Table 1 (DOCX 25 kb)

Supplementary Online Resource Table 2 (DOCX 23 kb)

Supplementary Online Resource Table 3 (DOCX 16 kb)

Supplementary Online Resource Table 4 (XLS 107 kb)

Supplementary Online Resource Table 5 (XLSX 790 kb)
