# Supplementary material for: miR-331-3p is involved in glucocorticoid resistance reversion by rapamycin through suppression of the MAPK signaling pathway
Source: Cancer Chemother Pharmacol. 2020 Aug 10;86(3):361–74. doi: 10.1007/s00280-020-04122-z (PMC7479018; doi:10.1007/s00280-020-04122-z)
Supplement: Supplementary file 1 — Supplementary Online Resource Fig.1 (DOCX 105 kb) [file 280_2020_4122_MOESM1_ESM.docx]

**Online Resource Figure 1**

Hierarchical clustering of samples based on the differentially expressed miRNAs in each experimental condition. Methylprednisolone (MP), rapamycin (RAPA).

MP+RAPA

MP

RAPA
