# Supplementary material for: miR-331-3p is involved in glucocorticoid resistance reversion by rapamycin through suppression of the MAPK signaling pathway
Source: Cancer Chemother Pharmacol. 2020 Aug 10;86(3):361–74. doi: 10.1007/s00280-020-04122-z (PMC7479018; doi:10.1007/s00280-020-04122-z)
Supplement: Supplementary file 2 — Supplementary Online Resource Table 1 (DOCX 25 kb) [file 280_2020_4122_MOESM2_ESM.docx]

Online Resource Table 1

Differentially expressed miRNAsafter treatment with methylprednisolone (MP), rapamycin (RAPA) and in combination (MP+RAPA). In bold are the miRNAs up- or down-regulated specifically during the co-treatment.

| **miRNA** | ***Relative Expression*** | | |
| --- | --- | --- | --- |
|  | **MP** | **RAPA** | **MP+RAPA** |
| hsa-let-7a | 1.00 | 2.80 | 2.21 |
| hsa-let-7d-5p | 1.00 | 7.09 | 4.21 |
| hsa-let-7e-5p | 1.00 | 1.77 | 2.29 |
| hsa-miR-101-3p | 97.84 | 16.06 | 36.31 |
| hsa-miR-107 | 0.05 | 1.00 | 2.03 |
| hsa-miR-125a-5p | 1.94 | 0.02 | 1.65 |
| hsa-miR-126-3p | 1.00 | 1.55 | 1.59 |
| hsa-miR-128a-3p | 1.00 | 2.33 | 2.32 |
| hsa-miR-130a-3p | 5.55 | 10.12 | 9.31 |
| hsa-miR-130b-3p | 1.00 | 2.75 | 1.97 |
| hsa-miR-133a-3p | 7.15 | 9.35 | 0.34 |
| hsa-miR-135b-3p | 1.00 | 80.37 | 3.01 |
| hsa-miR-138-5p | 1.00 | 0.45 | 0.01 |
| hsa-miR-139-5p | 0.04 | 0.07 | 2.88 |
| hsa-miR-140-3p | 1.00 | 1.51 | 1.00 |
| hsa-miR-141-3p | 23.21 | 23.40 | 3.01 |
| hsa-miR-142-5p | 1.00 | 0.37 | 1.00 |
| hsa-miR-146a-5p | 2.15 | 2.24 | 3.22 |
| hsa-miR-146b-5p | 1.60 | 1.60 | 1.84 |
| hsa-miR-147b | 1.00 | 114.01 | 3.01 |
| hsa-miR-148a-3p | 1.91 | 2.61 | 2.36 |
| hsa-miR-148b-3p | 3.77 | 7.51 | 1.54 |
| hsa-miR-149-5p | 3.07 | 6.39 | 0.05 |
| hsa-miR-150-5p | 1.75 | 3.14 | 5.58 |
| hsa-miR-152-3p | 0.01 | 0.02 | 0.03 |
| hsa-miR-155-5p | 1.74 | 2.34 | 2.72 |
| hsa-miR-15a-5p | 0.30 | 0.06 | 0.20 |
| hsa-miR-15b-5p | 1.00 | 2.17 | 2.57 |
| hsa-miR-181a-5p | 1.00 | 1.92 | 3.72 |
| hsa-miR-181c-5p | 0.44 | 1.00 | 1.00 |
| hsa-miR-182-5p | 2.14 | 4.87 | 7.07 |
| hsa-miR-183-5p | 0.41 | 0.19 | 1.00 |
| hsa-miR-185-5p | 7.86 | 7.93 | 12.45 |
| hsa-miR-192-5p | 0.40 | 1.00 | 1.00 |
| hsa-miR-194-5p | 0.01 | 1.00 | 0.03 |
| hsa-miR-197-3p | 1.00 | 3.34 | 4.83 |
| hsa-miR-199a-3p | 1.00 | 0.40 | 0.31 |
| **hsa-miR-19a-3p** | 1.00 | 1.00 | 0.50 |
| hsa-miR-200b-3p | 2.43 | 1.00 | 1.00 |
| hsa-miR-203-3p | 51.84 | 2.08 | 3.01 |
| hsa-miR-20a-5p | 1.00 | 2.14 | 1.55 |
| hsa-miR-21-5p | 1.00 | 0.25 | 1.53 |
| hsa-miR-210-3p | 1.00 | 0.19 | 0.27 |
| hsa-miR-212-3p | 5.17 | 3.45 | 4.82 |
| hsa-miR-219-1-3p | 26.27 | 31.05 | 35.06 |
| hsa-miR-221-3p | 2.70 | 4.56 | 5.93 |
| hsa-miR-222-3p | 1.00 | 1.68 | 2.36 |
| hsa-miR-223-3p | 2.24 | 2.27 | 3.57 |
| hsa-miR-25-3p | 1.00 | 2.47 | 1.84 |
| hsa-miR-26a-5p | 1.87 | 3.13 | 2.81 |
| hsa-miR-26b-5p | 1.00 | 1.97 | 1.00 |
| hsa-miR-27a-3a | 1.00 | 1.85 | 5.01 |
| hsa-miR-27b-3p | 0.01 | 1.00 | 0.01 |
| hsa-miR-28-5p | 1.00 | 2.93 | 1.00 |
| hsa-miR-28-3p | 1.00 | 1.74 | 2.13 |
| hsa-miR-29b-3p | 33.20 | 2.08 | 3.01 |
| hsa-miR-302a-3p | 0.35 | 0.20 | 1.00 |
| hsa-miR-302c-3p | 0.06 | 0.00 | 0.08 |
| **hsa-miR-30b-5p** | 1.00 | 1.00 | 1.69 |
| **hsa-miR-30c-5p** | 1.00 | 1.00 | 1.65 |
| hsa-miR-32-5p | 0.28 | 0.44 | 1.00 |
| hsa-miR-324-3p | 0.50 | 1.00 | 1.00 |
| hsa-miR-324-5p | 1.00 | 2.02 | 1.00 |
| hsa-miR-328-3p | 1.00 | 5.71 | 11.36 |
| hsa-miR-330-3p | 2.71 | 3.09 | 2.86 |
| hsa-miR-330-5p | 0.04 | 0.08 | 0.12 |
| **hsa-miR-331-3p** | 1.00 | 1.00 | 1.77 |
| hsa-miR-331-5p | 1.00 | 0.06 | 0.08 |
| hsa-miR-339-5p | 1.00 | 0.36 | 1.65 |
| hsa-miR-342-3p | 1.00 | 1.86 | 2.17 |
| **hsa-miR-345-5p** | 1.00 | 1.00 | 2.05 |
| hsa-miR-346 | 0.27 | 0.00 | 0.00 |
| hsa-miR-361-5p | 0.19 | 1.00 | 1.00 |
| hsa-miR-362-3p | 0.17 | 0.02 | 1.00 |
| hsa-miR-365a-3p | 1.00 | 0.33 | 1.00 |
| hsa-miR-367-3p | 1.00 | 0.37 | 0.48 |
| hsa-miR-374a-5p | 6071.45 | 5487.42 | 4857.82 |
| hsa-miR-422a | 1.82 | 1.00 | 1.55 |
| hsa-miR-423-5p | 2.82 | 2.58 | 9.46 |
| hsa-miR-424-5p | 0.03 | 0.05 | 2.50 |
| hsa-miR-450b-5p | 0.09 | 21.74 | 0.24 |
| hsa-miR-454-3p | 1.00 | 1.72 | 1.00 |
| hsa-miR-455-5p | 0.01 | 1.00 | 1.00 |
| hsa-miR-455-3p | 1.00 | 0.15 | 1.00 |
| hsa-miR-483-5p | 0.21 | 0.18 | 0.35 |
| hsa-miR-487a-3p | 0.34 | 0.06 | 1.00 |
| hsa-miR-500a-5p | 2.31 | 3.39 | 1.56 |
| hsa-miR-501-5p | 1.00 | 0.40 | 1.00 |
| hsa-miR-502-3p | 4.34 | 4.88 | 0.29 |
| hsa-miR-505-3p | 1.00 | 0.19 | 7.73 |
| hsa-miR-517b-3p | 0.49 | 0.09 | 1.00 |
| hsa-miR-518f-3p | 272.12 | 2.08 | 3.01 |
| hsa-miR-519a-3p | 1.00 | 2.08 | 20671.62 |
| hsa-miR-522-3p | 23492.44 | 2.08 | 4339.22 |
| hsa-miR-523-3p | 52.77 | 2.08 | 7429.95 |
| hsa-miR-532-5p | 1193.71 | 2.08 | 609.87 |
| hsa-miR-532-3p | 833.87 | 2.08 | 2340.30 |
| hsa-miR-542-3p | 2.28 | 2.16 | 0.16 |
| hsa-miR-542-5p | 1.00 | 2.08 | 92.65 |
| hsa-miR-548a-3p | 1.00 | 64.46 | 3.01 |
| hsa-miR-548d-5p | 0.24 | 0.37 | 12.65 |
| hsa-miR-574-3p | 6.30 | 2.62 | 2.50 |
| hsa-miR-576-3p | 0.01 | 1.00 | 1.00 |
| hsa-miR-579 | 1.00 | 73.11 | 3.01 |
| hsa-miR-590-5p | 4489.20 | 3673.70 | 1908.54 |
| hsa-miR-616-5p | 1.00 | 2.08 | 67.03 |
| hsa-miR-618 | 1693.82 | 963.47 | 24523.69 |
| hsa-miR-625-5p | 115.24 | 195.27 | 225.15 |
| hsa-miR-627 | 2377.58 | 3131.03 | 2076.76 |
| hsa-miR-628-5p | 9887.29 | 1902.38 | 103373.26 |
| hsa-miR-636 | 161.02 | 309.80 | 429.06 |
| hsa-miR-642-5p | 75.71 | 95.17 | 10.15 |
| hsa-miR-652-3p | 201.23 | 675.03 | 507.16 |
| hsa-miR-660-5p | 1333.54 | 615.23 | 653.08 |
| **hsa-miR-744-5p** | 1.00 | 1.00 | 1.58 |
| **hsa-miR-886-3p** | 1.00 | 1.00 | 0.10 |
| **hsa-miR-886-5p** | 1.00 | 1.00 | 0.24 |
| hsa-miR-92a-3p | 14131.77 | 26428.08 | 37950.82 |
