# Supplementary material for: miR-331-3p is involved in glucocorticoid resistance reversion by rapamycin through suppression of the MAPK signaling pathway
Source: Cancer Chemother Pharmacol. 2020 Aug 10;86(3):361–74. doi: 10.1007/s00280-020-04122-z (PMC7479018; doi:10.1007/s00280-020-04122-z)
Supplement: Supplementary file 3 — Supplementary Online Resource Table 2 (DOCX 23 kb) [file 280_2020_4122_MOESM3_ESM.docx]

Online Resource Table 2.

MiRpath analyses: miRNAs potentially involved in the deregulation of MAPK and ErbB signaling pathway and relatives target genes.

| MAPK signaling pathway | |
| --- | --- |
| miRNAs | **n° Target Genes** |
| hsa-miR-422a | 2 |
| hsa-miR-331-3p | 8 |
| hsa-miR-423-5p | 13 |
| hsa-miR-107 | 6 |
| hsa-miR-222-3p | 4 |
| hsa-miR-141-3p | 3 |
| hsa-miR-182-5p | 3 |
| hsa-miR-125a-5p | 4 |
| hsa-miR-342-3p | 2 |
| hsa-miR-223-3p | 1 |
| hsa-miR-328-3p | 6 |
| hsa-miR-625-5p | 7 |
| hsa-miR-424-5p | 9 |
| hsa-miR-15b-5p | 9 |
| hsa-miR-542-5p | 8 |
| hsa-miR-197-3p | 4 |
| hsa-miR-101-3p | 3 |
| hsa-miR-532-3p | 4 |
| hsa-miR-185-5p | 3 |
| hsa-let-7e-5p | 2 |
| hsa-let-7d-5p | 2 |
| hsa-miR-221-3p | 1 |
| hsa-miR-590-5p | 2 |
| hsa-miR-505-3p | 2 |
| hsa-miR-28-3p | 1 |
| hsa-miR-532-5p | 2 |
| hsa-miR-519a-3p | 1 |
| hsa-miR-548a-3p | 1 |
| hsa-miR-29b-3p | 2 |
| hsa-miR-25-3p | 1 |
| hsa-miR-92a-3p | 1 |
| hsa-miR-518f-3p | 1 |
| hsa-miR-135b-3p | 3 |
| hsa-miR-148b-3p | 3 |
| hsa-miR-130b-3p | 1 |
| hsa-miR-148a-3p | 3 |
| hsa-miR-130a-3p | 1 |
| hsa-miR-146b-5p | 1 |
| hsa-miR-146a-5p | 1 |
| hsa-miR-21-5p | 1 |
| hsa-miR-339-5p | 2 |

| MAPK signalingpathway | |
| --- | --- |
| List of Genes | |
| CACNA1I | MAP2K4 |
| FGF11 | TNF |
| AKT2 | RELA |
| NLK | CACNA1D |
| DUSP10 | NFATC3 |
| RASGRP1 | FGFR1 |
| MAPK8 | DUSP16 |
| TRAF6 | FGF16 |
| MAPKAPK3 | FGF7 |
| PDGFB | RAP1B |
| PRKACA | TGFBR2 |
| CACNB1 | ELK1 |
| TRAF2 | PLA2G4C |
| FLNA | ARRB1 |
| CACNA1E | PRKACB |
| MAPT | RPS6KA2 |
| CACNG4 | AKT3 |
| SOS1 | MAPK8IP3 |
| DUSP8 | FGF8 |
| FGF2 | STMN1 |
| FGF12 | FGF10 |
| NTRK2 | PPP3R1 |
| NTF3 | CHUK |
| CACNG8 | MAP4K3 |
| PDGFRA | RPS6KA1 |
| TGFBR1 | RASGRP2 |
| CACNA2D3 | GADD45A |
| NFKB1 | MAP3K13 |
| SOS2 | DUSP7 |
| MAP4K2 | FAS |
| ATF2 | RPS6KA3 |
| RAC2 | TGFB2 |
| PPP5C | CACNA1H |
| FGFR3 | MRAS |
| ELK4 | MAP3K4 |
| CRK | FASLG |
| MAPK7 | DUSP5 |
| MAP2K7 | HSPA1B |
| CACNB4 | TAOK1 |
| FGF5 | MAP2K6 |
| RASGRF2 | RAP1A |
| DUSP6 | CACNA2D4 |
| MAPK14 | MKNK1 |
| FGF18 | KRAS |
| CACNA2D2 | CACNG7 |
| CACNA2D1 | CDC42 |
| CACNB2 | AC008687.1 |
| BDNF | CACNA1S |
| MKNK2 | HSPA1L |

| ErbBsignalingpathway | |
| --- | --- |
| miRNAs | **n° Target Genes** |
| hsa-miR-138-5p | 4 |
| hsa-miR-149-5p | 1 |
| hsa-miR-152-3p | 4 |
| hsa-miR-19a-3p | 1 |
| hsa-miR-331-5p | 1 |
| hsa-miR-199a-3p | 1 |
| hsa-miR-27b-3p | 3 |
| hsa-miR-367-3p | 2 |
| hsa-miR-330-5p | 1 |
| hsa-miR-302c-3p | 2 |
| hsa-miR-346 | 1 |
| hsa-miR-483-5p | 1 |
| hsa-miR-15a-5p | 1 |
| hsa-miR-194-5p | 1 |

| ErbBsignalingpathway | |
| --- | --- |
| List of Genes | |
| GSK3B | GAB1 |
| HBEGF | SHC4 |
| SOS2 | AKT3 |
| PIK3CB | PAK6 |
| TGFA | PIK3CA |
| EIF4EBP1 | MAP2K4 |
| KRAS | GRB2 |
| PAK3 | ELK1 |
| STAT5B | SOS1 |
| CBLB | MAPK3 |
| PIK3R3 | PAK6 |
