# Supplementary material for: miR-331-3p is involved in glucocorticoid resistance reversion by rapamycin through suppression of the MAPK signaling pathway
Source: Cancer Chemother Pharmacol. 2020 Aug 10;86(3):361–74. doi: 10.1007/s00280-020-04122-z (PMC7479018; doi:10.1007/s00280-020-04122-z)
Supplement: Supplementary file 4 — Supplementary Online Resource Table 3 (DOCX 16 kb) [file 280_2020_4122_MOESM4_ESM.docx]

| TargetScan Predicted Interactions for hsa-miR-331-3p | |
| --- | --- |
| GENE | **Context Score** |
| CACNG8 | -0.757 |
| MAP4K2 | -0.426 |
| MAP2K7 | -0.736 |
| FGF11 | -0.425 |
| CACNB1 | -0.852 |
| DUSP5 | -0.563 |
| TNF | -0.407 |
| CACNA1D | -0.630 |

Online Resource Table 3.

Putative targets genes ofmiR-331-3p predicted by Diana-TargetScan.
